# Velutin Inhibits IL-1β-Induced Nucleus Pulposus Inflammatory and Extracellular Matrix Degradation Attenuating Mouse Intervertebral Disc Degeneration via the NF-κB and MAPK Pathways

**DOI:** 10.1155/mi/9625485

**Published:** 2025-11-20

**Authors:** Yufeng Zhang, Rongjian Xu, Kelei Wang, Xinyu Li, Yun Lou, Li Cao, Wanlei Yang, Yu Qian

**Affiliations:** ^1^Department of Orthopedics, Shaoxing People's Hospital (Shaoxing Hospital, Zhejiang University School of Medicine), Shaoxing 312000, Zhejiang, China; ^2^Xianju County Peoples Hospital, Taizhou 318000, Zhejiang, China; ^3^Center for Rehabilitation Medicine, Department of Orthopedics, Zhejiang Provincial People's Hospital (Affliated People's Hospital), Hangzhou Medical College, Hangzhou, Zhejiang, China; ^4^Department of Orthopedics Surgery, The First Affiliated Hospital of Zhejiang Chinese Medical University (Zhejiang Provincial Hospital of Chinese Medicine), Hangzhou 310006, Zhejiang, China

**Keywords:** intervertebral disk degeneration, mitogen-activated protein kinase, nuclear factor-**κ**B, velutin

## Abstract

Intervertebral disk degeneration (IVDD) usually causes lower back pain (LBP). Mechanical stress, trauma, aseptic inflammation, infection, and genetic susceptibility can accelerate the development of IVDD. Reportedly, proinflammatory cytokines and extracellular matrix (ECM) degradation play significant roles in IVDD progression. Therefore, anti-inflammatory treatments and ECM inhibition can potentially delay the progression of IVDD. Velutin, a natural flavonoid, has the vigorous effects of suppressing inflammation. In this study, we researched the protective effects of velutin and the underlying mechanisms. Additionally, we evaluated its validity in a mouse IVDD model. The results indicated that velutin effectively suppressed interleukin-1β-induced inflammatory mediators. Moreover, our findings revealed the mechanisms of velutin's anti-inflammatory effects. Our results indicate that velutin is a potential therapeutic agent for IVDD.

## 1. Introduction

Lumbar intervertebral disc degeneration (IVDD) is the major underlying cause of lower back pain (LBP), and its progression leads to degenerative spinal disorders, such as spinal stenosis, which impose an enormous economic burden on society [1, 2]. The occurrence and development of IVDD are caused by abnormal mechanical stress, trauma, infection, genetic susceptibility, inflammation, and reduced local tissue nutrient transport [3].

Intervertebral discs (IVDs) contain nucleus pulposus (NP) cells that secrete extracellular matrix (ECM) [4]. The ECM performs critical functions in alleviating the axial mechanical pressure on the body and maintaining disc height. Inflammation, senescence, and ECM degradation induced by NPs are the primary molecular mechanisms underlying IVDD [5]. Cellular senescence and inflammatory responses contribute to the increased breakdown of NPs, leading to ECM degradation [6].

Interleukin 1 beta (IL-1β) is the crucial inflammatory factor of NPs [7]. As a result, matrix metalloproteinases (MMPs) and A disintegrin and metalloproteinase with thrombospondin motifs (ADAMTS) increase, leading to loss of the ECM and development of IVDD [8]. Therefore, effective inhibition of inflammatory mediators can delay the progression and treatment of IVDD. Nuclear factor-κB (NF-κB) and mitogen-activated protein kinase (MAPK) signaling pathways are considered the main inflammatory responses to IL-1β stimulation in NPs [9]. These pathways create a vicious spiral of inflammatory cascades that lead to the progression and occurrence of IVDD [10, 11].

Velutin, a naturally occurring flavonoid compound, exhibits significant therapeutic potential as a dual anti-inflammatory and disease-modifying pharmacological agent. Its mechanism-driven bioactivity positions it as a promising candidate for the management of diverse inflammatory-mediated pathological conditions [12]. We previously found that velutin can exert a dual protective effect by inhibiting subchondral bone loss and delaying cartilage degradation in osteoarthritis [13]. Velutin effectively suppresses the expression of proinflammatory cytokines [14, 15]. While velutin's anti-inflammatory efficacy has been extensively documented in preclinical investigations, demonstrating marked efficacy in attenuating pro-inflammatory cascades, its therapeutic implications for IVDD remain insufficiently characterized within the specific pathomechanistic context of discogenic pathology [16].

In our study, we designed an experiment of inflammation in NPs and investigated the resistance of inflammatory effects by velutin. We studied the mechanisms of its anti-inflammatory effects. We used a mouse model of spinal instability to confirm the therapeutic efficacy of velutin for IVDD.

## 2. Materials and Methods

### 2.1. Reagents

We purchased velutin from ChemFace (Wuhan, Hubei, China). The primary antibodies targeting ADAMTS5 (ab41037), collagen II (ab34712) and collagen X (ab58632) were sourced from Abcam (Cambridge, UK). Primary antibodies against p-ERK (#4370), p-JNK (#4668), p-p65 (#3033), p-IκBα (#2859), ERK (#4695), JNK (#9258), p65 (#8242), IκBα (#4814), COX-2 (#12282), iNOs (#13120), and MMP13 (#69926) were purchased from Cell Signaling Technology (Danvers, MA, USA). Gibco (Rockville, MD, USA) supplied trypsin-EDTA (0.25%), fetal bovine serum, DMEM/F12, streptomycin, and penicillin.

### 2.2. Mouse NP Isolation and Culture

Mouse NPs were isolated from young C57-BL mice (male, average weight of ~25 g). Briefly, lumbar vertebral tissues were collected, and NP tissues were placed in a solution of 0.25% type II collagenase prepared with DMEM/F12 medium with continuous shaking for 1.5 h. The cells were then cultivated for 1 week. Cells from the second passage were used for subsequent experiments.

### 2.3. Cytotoxicity Assay

To evaluate the effects of velutin on NP cells, cells were seeded in 96-well plates at 5 × 10^3^ cells/well. Peripheral wells containing complete medium without cells served as blank controls. NP cells were treated with velutin at graded concentrations (0, 2, 4, 8, 16, 32, 64, 128 μM) in complete medium for 24 h or 48 h. Culture medium was replaced with 100 μL fresh medium containing 10% CCK-8 reagent. Following 1.5 h incubation under standard conditions, liquid surface bubbles were eliminated by gentle airflow. Plates were agitated for 5 min on an orbital shaker (50 rpm) to ensure homogeneous color development. Absorbance was measured using a microplate reader, data represent triplicate independent experiments.

### 2.4. Western Blotting Assay

The NPs were seeded at a density of 2 × 10^5^ cells in 6-well plates for 24 h. The cells were subsequently divided into experimental groups: one group received IL-1β (10 ng/mL) stimulation alone, while the other group was co-treated with IL-1β (10 ng/mL) and velutin (4, 8, 16, and 32 μM) for 24 h.

Total protein extraction was performed using RIPA lysis buffer containing protease inhibitors. Following 20-min incubation on ice, proteins were centrifuged at 13,300 rpm for 15 min at 4°C. Proteins were determined using a BCA protein assay kit. Equal amounts of proteins were mixed with sodium dodecyl sulfate sampling buffer and denatured at 95°C for 10 min. Proteins were separated by 10% SDS-PAGE and transferred to PVDF membranes. After blocking with 5% non-fat milk in TBST for 1 h, proteins were incubated overnight at 4°C with primary antibodies against target proteins. Following three TBST washes, proteins were probed with HRP-conjugated secondary antibodies for 1 h. Proteins were visualized using enhanced chemiluminescence and quantified by densitometry using Image Lab software.

### 2.5. Real-Time Polymerase Chain Reaction

The NPs were seeded at a density of 2 × 10^5^ cells in 6-well plates for 24 h. Total RNA was extracted from NP cells using the RNAeasy Animal RNA Extraction Kit (Beyotime Biotechnology, Shanghai, China; Cat# R0026) following the manufacturer's protocol. RNA concentration and purity were determined by spectrophotometric analysis. For reverse transcription, 1 μg of total RNA was converted to cDNA using PrimeScript RT Master Mix (Takara Bio, Shiga, Japan; Cat# RR036A) under the following conditions: 37°C for 15 min and 85°C for 5 s. Quantitative PCR was performed using SYBR Green Premix Ex Taq (Takara Bio; Cat# RR420A) on a Bio-Rad CFX96 Real-Time PCR System. Reaction mixtures (20 μL) contained 1 μL cDNA template, 10 μM forward/reverse primers, and 10 μL SYBR Green Master Mix. Thermal cycling parameters: initial denaturation at 95°C for 10 min, followed by 40 cycles of 95°C for 10 s, 60°C for 20 s, and 72°C for 20 s, with a final extension at 72°C for 90 s. Melting curve analysis confirmed primer specificity. All reactions were performed in triplicate with no-template controls included in each run. The specific sequences of the mouse primers used are listed in Table 1.

### 2.6. Immunofluorescence (IF) Microscopy

NPs were seeded at a density of 3 × 10^3^ cells/well in 24-well plates, and the remaining steps were performed as described previously [13]. The primary antibody against COX-2 was diluted to 1:200.

### 2.7. Animal Experiments

Animal experiments strictly followed the principles of the *Guide for the Care and Use of Laboratory Animals* (NIH publication number 85–23, revised 1996) and were approved by the Animal Ethical Committee of Shaoxing People's Hospital, Shaoxing, Zhejiang Province, China (No. 2022Z032).

To establish the lumbar instability model, a depilating cream was used to remove hair from wild-type C57BL/6 mice. Subsequently, the spinous process and paraspinal muscles were exposed using a surgical scalpel. Ophthalmic scissors were used to excise the bilateral paravertebral muscles to expose the spinous processes. The spinous processes of L1–L4 were excised using ophthalmic scissors; in the control group, only the skin was incised. Rigorous disinfection and hemostasis were ensured, and the operative site was sutured using nylon sutures. All procedures were performed under general anesthesia using 3% isoflurane inhalation. Once the model was successfully established, mice were intraperitoneally injected with velutin (at doses of 5 or 10 mg/kg, 100 µL) or PBS (100 µL) daily. Four weeks postsurgery, the mice were euthanized, and their lumbar spinal tissues, along with other tissues, were harvested for further study. Throughout the study, all mice were maintained in a pathogen-free environment.

### 2.8. Histopathological Analysis

Mice were euthanized 5 weeks after surgery. Euthanasia was performed using an excessive dose of 4% pentobarbital, and the lumbar regions were collected. Subsequently, specimens were decalcified, fixed in formaldehyde, dehydrated, and embedded in paraffin. The resulting tissue samples were sectioned into 5-μm slices. Individual disc slides were stained with SO-FG and hematoxylin as HE staining.

### 2.9. Statistical Analysis

All experiments were repeated three times, and the data are presented as the mean ± standard deviation (SD). Two-tailed Student's *t* tests were used to assess significant differences using SPSS 21.0 (Chicago, IL, USA).

## 3. Results

### 3.1. Velutin Suppresses the Production of Inflammatory Factors in IL-1β-Induced NPs In Vitro

**Figure 1:**
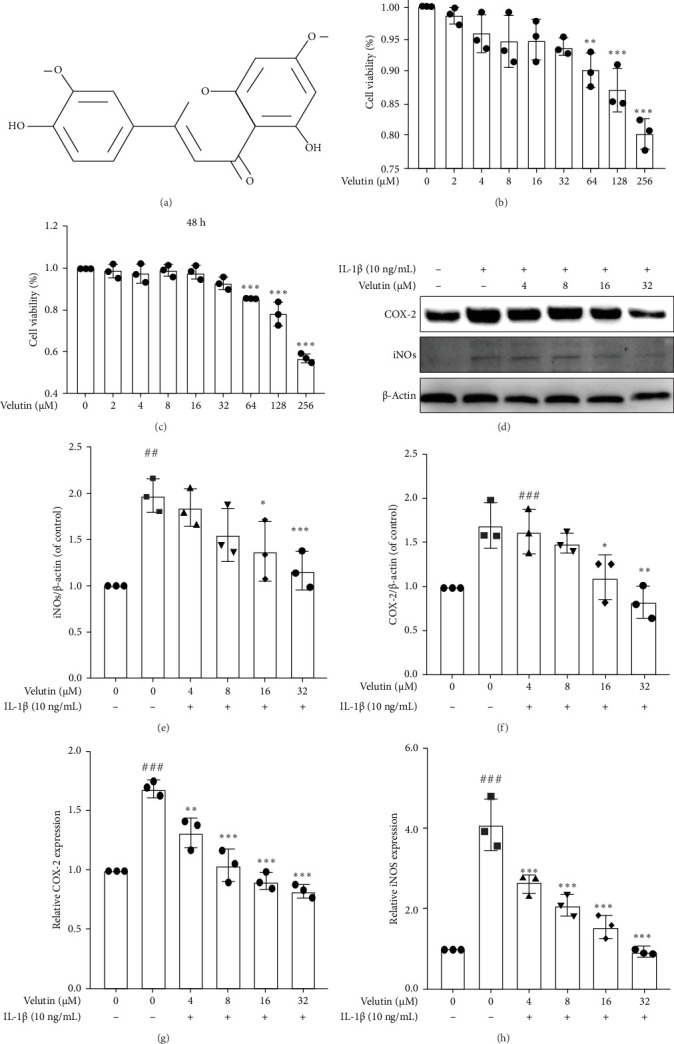
Velutin inhibits the IL-1β-induced inflammatory response in NPs. (A) Chemical structure of velutin. (B, C) Cell viability of velutin. (D–F) Protein expression levels of iNOs and COX-2. (G, H) Gene expression levels of COX-2 and iNOs. Data are presented as the mean ± standard deviation (SD); ###*p* < 0.001 vs. control group; *⁣*^*∗*^*p* < 0.05, *⁣*^*∗∗*^*p* < 0.01, and *⁣*^*∗∗∗*^*p* < 0.01 vs. IL-1β alone (*n* = 3). Vel, velutin.

The molecular structure of velutin is shown in Figure 1A. The results from NPs cultured for 24 and 48 h demonstrated that velutin at 0–32 μM exhibits nontoxicity (Figure 1B, C). However, velutin concentrations of 64–256 μM caused increasing cytotoxicity to NPs as the concentration increased. Therefore, for subsequent experiments, we used velutin at concentrations of 0–32 μM to treat the NPs. The expression levels increased in the IL-1β group (Figure 1), while velutin dose-dependently reduced their expression. Furthermore, changes in gene expression levels were similar to those determined by qPCR (Figure 1G, H). These results indicate that velutin can inhibit the inflammatory response in NPs.

### 3.2. Velutin Mitigates IL-1β-Induced ECM Degradation in NPs

**Figure 2:**
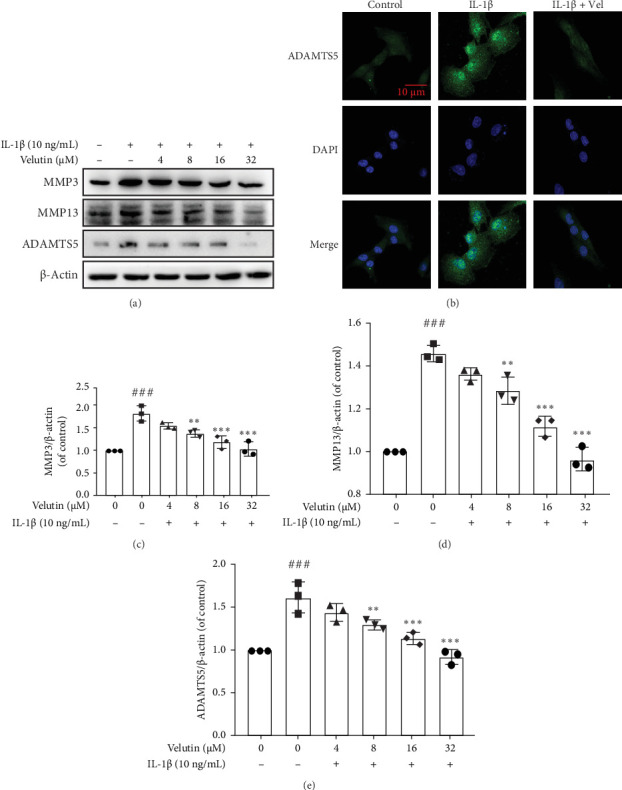
Velutin suppresses the expression of ECM-degrading enzymes in NPs. (A, C–E) Protein expression levels and quantification of MMP3, MMP13, and ADAMTS5 in NPs. (B) IF was performed to measure the fluorescence intensity of ADAMTS5 in NPs. The velutin concentration used was 32 μM. Scale bar: 10 μm. Data are presented as the mean ± standard deviation (SD); ###*p* < 0.001 vs. control group; *⁣*^*∗*^*p* < 0.05 and *⁣*^*∗∗∗*^*p* < 0.01 vs. interleukin-1 beta (IL-1β) alone (*n* = 3). Vel, velutin.

Studies have shown that MMPs and ADAMTs are among the key proteases involved in the ECM degradation of NPs [8]. We quantitatively analyzed the expression levels of these proteins (Figure 2A, C, D). MMP3, MMP13, and ADAMTS5 were upregulated, whereas velutin reduced the expression levels of these proteins. IF analysis of the ADAMTS5 fluorescence intensity in different groups (Figure 2B) revealed that IL-1β decreased the fluorescence intensity of Collagen Ⅱ, while velutin increased the fluorescence intensity. These results indicate that velutin inhibits ECM degradation.

### 3.3. Velutin Enhances the Synthesis of ECM in NPs

**Figure 3:**
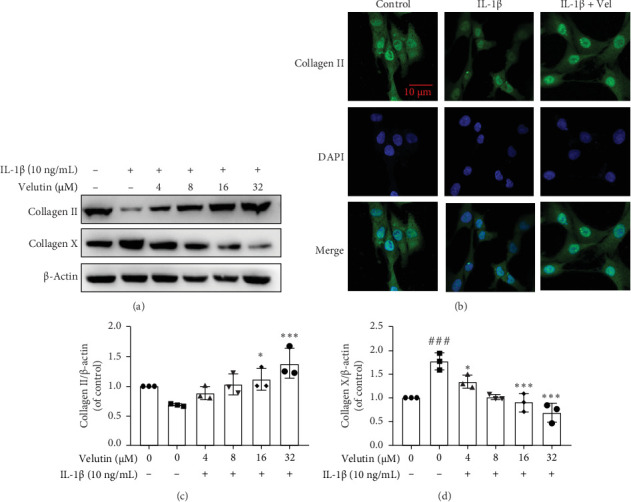
Velutin promotes the synthesis of the ECM in NPs. (A, C, D) Protein expression levels and quantitative analysis of collagen Ⅱ and collagen X in NPs. (B) IF was performed to evaluate the effect of IL-1β on collagen Ⅱ in NPs. The velutin concentration used was 32 μM. Scale bar: 10 μm. Data are presented as the mean ± standard deviation (SD); ###*p* < 0.001 vs. control group; *⁣*^*∗∗*^*p* < 0.01 and *⁣*^*∗∗∗*^*p* < 0.01 vs. IL-1β alone (*n* = 3). Vel, velutin.

NPs, which are chondrocyte-like cells, are the primary cells in the IVD. They play a crucial role in maintaining the stability and function of the IVD. Collagen Ⅹ is a marker protein for cell hypertrophy. We examined the expression levels of the relevant proteins. Collagen II expression increased in a dose-dependent manner in the presence of velutin (Figure 3A). However, collagen X expression increased with IL-1β stimulation, and velutin treatment reversed this effect (Figure 3A). IF revealed a noticeable decrease in the fluorescence intensity of collagen Ⅱ under IL-1β stimulation, which was increased with velutin (Figure 3B). These findings indicate that velutin promotes ECM synthesis in NPs.

### 3.4. Velutin Inhibits the NF-κB and MAPK Signaling Pathways to Attenuate the IL-1β-Induced Inflammatory Response

**Figure 4:**
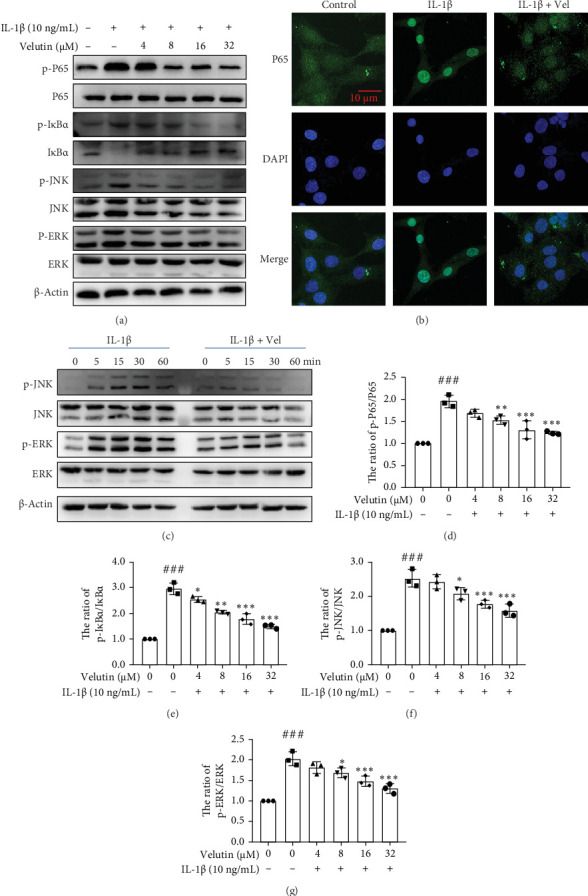
Velutin inhibits IL-1β-induced activation of the NF-κB and MAPK pathways. (A, D, E, F, G) Protein expression levels and quantitative analysis of the NF-κB and MAPK pathways were examined. (B) IF was performed to visualize the subcellular localization of p65. The velutin concentration used was 32 μM. Scale bar: 10 μm. (C) The inhibitory effect of velutin on the MAPK pathway at different time points of stimulation was assessed. Data are presented as the mean ± standard deviation (SD); ###*p* < 0.001 vs. control group; *⁣*^*∗*^*p* < 0.05, *⁣*^*∗∗*^*p* < 0.01, and *⁣*^*∗∗∗*^*p* < 0.01 vs. IL-1β alone (*n* = 3).

To explore the potential protective mechanisms of velutin against the inflammatory response in NPs, we analyzed the protein expression of p-IκBα, p-p65, IκBα, and p65 (Figure 4A, D, E). IL-1β stimulation significantly increased the ratio of p-p65/p65, upregulated p-IκBα expression, downregulated IκBα expression, and increased the ratio of p-IκBα/IκBα. Velutin treatment inhibited the phosphorylation and degradation of IκBα. Additionally, IF revealed that after IL-1β stimulation, p65 was translocated from the cytoplasm to the nucleus, while velutin treatment partially reversed this effect (Figure 4B). As indicated by the experimental results, under IL-1β stimulation, the ratios of p-JNK/JNK and p-ERK/ERK were significantly increased, whereas velutin treatment reduced these ratios (Figure 4A, F, G). We also observed that compared with IL-1β stimulation, velutin caused the most significant reduction in the p-JNK/JNK ratio at 15 min of stimulation and a noticeable decrease in the p-ERK/ERK ratio at 5 min (Figure 4C). Therefore, the data suggest that velutin exerts a protective effect on NPs by inhibiting the activation of the NF-κB and MAPK pathways induced by IL-1β.

### 3.5. Velutin Protects Against IVDD In Vivo

**Figure 5:**
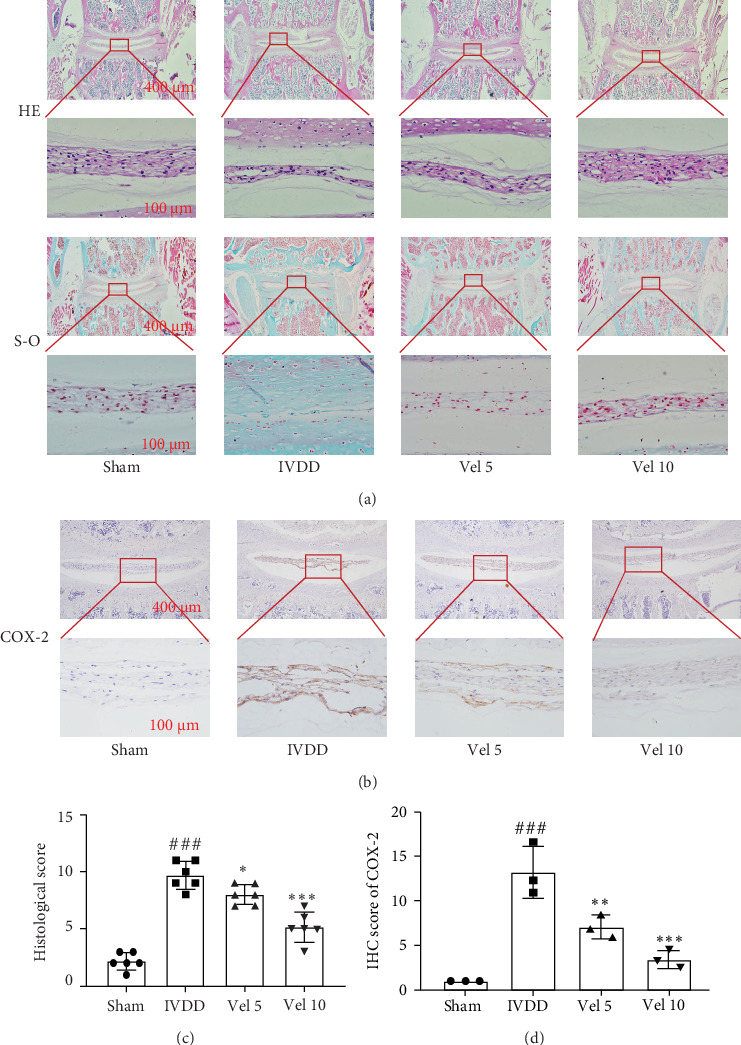
Velutin alleviates progression in a mouse IVDD model. (A) HE staining and Safranin-O (S-O)/Fast Green staining of NP tissue. (B, D) Immunohistochemical staining and quantitative analysis of COX-2 in intervertebral disc sections. (C) Histological scoring of IVDD. Scale bar: 10 μm. Data are presented as the mean ± SD; ###*p* < 0.001 vs. control group; *⁣*^*∗*^*p* < 0.05, *⁣*^*∗∗*^*p* < 0.01, and *⁣*^*∗∗∗*^*p* < 0.01 vs. interleukin-1 beta (IL-1β) alone (*n* = 3). Vel 5, 5 mg/kg velutin; Vel 10, 10 mg/kg velutin.

To investigate whether velutin delays the progression of IVDD in vivo, we conducted experiments using a mouse model of IVDD. First, a preliminary experiment was performed to determine the safe velutin dosage in mice. The experiment proper consisted of four groups: sham, IVDD, IVDD + low-dose (5 mg/kg) velutin, and IVDD + high-dose (10 mg/kg) velutin. After 4 weeks of treatment, lumbar spine samples were collected and subjected to HE and S-O/FG staining (Figure 5A). The IVDD group exhibited significant fibrous ring disruption, reduced or even absent NP tissue, and a narrowed intervertebral space. In the low-dose group, there was an increase in NP tissue, and the high-dose group showed an even more pronounced increase in NP tissue. The histological scores of the IVDD group (Figure 5C) were significantly increased, whereas velutin treatment reduced the histological scores. Immunohistochemical results (Figure 5B, D) showed upregulation of COX-2 expression in the IVDD group, which was reduced by velutin treatment. Therefore, these results demonstrate that velutin protects against IVDD in vivo and reduces the expression of COX-2.

## 4. Discussion

With a global aging population, the number of patients with IVDD is increasing, resulting in a significant economic burden [7]. The etiology of IVDD is complex and primarily involves genetic susceptibility, unhealthy lifestyle factors (such as obesity, smoking, and staying up late), and long-term abnormal pressure loads, which can lead to inflammatory reactions, cell apoptosis, and ECM metabolism imbalances in NPs [17, 18]. IL-1β is considered a vital proinflammatory cytokine that exerts its proinflammatory effect by producing various mediators [19]. Previous studies have reported that IL-1β is elevated in IVDD, and this effect is closely related to imbalances in the internal environment, leading to the degradation of NP tissue [20]. In our experiments, we used IL-1β to simulate the inflammatory process in NPs, mimicking the in vivo IVDD procedure.

Given the incomplete understanding of IVDD pathogenesis, current clinical therapeutic strategies primarily focus on symptomatic management. When disc degeneration results in neural compression manifestations, treatment modalities typically include pharmacologic interventions, physical rehabilitation techniques, peripheral nerve blockade procedures, and in refractory cases, surgical decompression [21]. Nonetheless, these therapies are unable to fundamentally halt or reverse IVDD advancement, underscoring the pressing need for an efficacious clinical treatment for IVDD.

As per our prior research, velutin, a small molecule derived from mistletoe, can effectively suppress the expression of proinflammatory cytokines [13]. Despite several studies demonstrating the anti-inflammatory properties of velutin, its specific role in IVDD remains unclear.

In our study, we proved the protective effects of velutin against IVDD and the underlying mechanisms. First, we determined safe concentrations of velutin for treating NP cells in vitro. Inflammation plays a crucial role in IVDD, as it can induce cellular senescence and apoptosis through inflammatory factors and lead to ECM degradation by activating MMPs and ADAMTSs [22]. We demonstrated that velutin lowered the expression of inflammatory mediators. MMPs and ADAMTSs directly contribute to the degradation of NP ECM, leading to the eventual breakdown of NPs and the progression of IVDD [23]. Our experiments demonstrated that velutin reduced the expression of ATAMTS5, MMP3, and MMP13 in NPs. Collagen X serves as a marker of chondrocyte hypertrophy and plays a role in ossification, while collagen II is a component of the ECM [24]. IL-1β leads to the destruction of the ECM by inhibiting the expression of collagen II and promotes the development of IVDD [25]. Our data show that velutin can help maintain the metabolic balance of the ECM in NPs.

In particular, the NF-κB and MAPK signaling pathways are believed to be involved in IL-1β-induced inflammation during the progression of IVDD [9]. Activation of the NF-κB signaling pathway can lead to an increase in various inflammatory phenotypes and further exacerbate cellular damage through positive feedback between inflammatory factors and NF-κB signaling activation [11]. NF-κB is a classical inflammatory-related pathway. The MAPK signaling pathway is closely associated with ECM degradation during IVDD progression, with increasing evidence [26]. In our study, velutin powerfully inhibited the activation of the IL-1β-induced MAPK and NF-κB pathways in NPs. Therefore, we believe that velutin exerts its protective effects through the MAPK and NF-κB signaling pathways.

Finally, we validated the protective effects of velutin on IVDD using a mouse model. Velutin increased the number of NPs, reduced the histological scores in the IVDD model [27], and decreased the expression of COX-2 in the IVD tissue. This implies that velutin could have a protective effect against IVDD in live subjects.

This is the first study to investigate the anti-inflammatory effects of velutin on IVDD in vitro and in vivo. However, the study had several limitations. First, in animal models, the effects of velutin on annulus fibrosus and cartilaginous endplate cells cannot be excluded. Second, the correlation between the NF-κB and MAPK pathways requires further exploration, as there might be a shared upstream regulator for these two signaling pathways, while the specific target of velutin remains unidentified. Third, the mouse model of IVDD has certain limitations and cannot fully mimic the process of IVDD in humans, moreover, the ECM could not be measured due to the experimental conditions. Further research is needed to determine the effects of velutin on IVDD.

In conclusion, our experiments demonstrate that velutin can alleviate IVDD by inhibiting the MAPK and NF-κB pathways in NPs and a mouse IVDD model. Our findings provide new and effective options for the treatment of IVDD.

## Figures and Tables

**Table 1:** Primer sequences for real-time polymerase chain reaction (RT‒PCR).

Genes	Forward primer (5′ to 3′)	Reverse primer (5′ to 3′)
β-actin COX-2 iNOs	AGCCATGTACGTAGCCATCC TGAGCAACTATTCCAAACCAGC GTTCTCAGCCCAACAATACAAGA	CTCTCAGCAGTGGTGGTGAA GCACGTAGTCTTCGATCACTATC GTGGACGGGTCGATGTCAC

## Data Availability

The data used to support the findings of this study are available from the corresponding author upon request.

## References

[1] Guterl C. C., See E. Y., Blanquer S. B. G. (2013). Challenges and Strategies in the Repair of Ruptured Annulus Fibrosus. *European Cells and Materials*.

[2] Blanquer S. B. G., Grijpma D. W., Poot A. A. (2015). Delivery Systems for the Treatment of Degenerated Intervertebral Discs. *Advanced Drug Delivery Reviews*.

[3] Binch A. L. A., Fitzgerald J. C., Growney E. A., Barry F. (2021). Cell-Based Strategies for IVD Repair: Clinical Progress and Translational Obstacles. *Nature Reviews Rheumatology*.

[4] Newell N., Little J. P., Christou A., Adams M. A., Adam C. J., Masouros S. D. (2017). Biomechanics of the Human Intervertebral Disc: A Review of Testing Techniques and Results. *Journal of the Mechanical Behavior of Biomedical Materials*.

[5] Lyu F.-J., Cheung K. M., Zheng Z., Wang H., Sakai D., Leung V. Y. (2019). IVD Progenitor Cells: A New Horizon for Understanding Disc Homeostasis and Repair. *Nature Reviews Rheumatology*.

[6] Nailwal N. P., Doshi G. M. (2021). Role of Intracellular Signaling Pathways and Their Inhibitors in the Treatment of Inflammation. *Inflammopharmacology*.

[7] Liu Z.-M., Lu C.-C., Shen P.-C. (2021). Suramin Attenuates Intervertebral Disc Degeneration by Inhibiting NF-κB Signalling Pathway. *Bone & Joint Research*.

[8] Zhu X., Liu S., Cao Z. (2021). Higenamine Mitigates Interleukin-1β-Induced Human Nucleus Pulposus Cell Apoptosis by ROS-Mediated PI3K/Akt Signaling. *Molecular and Cellular Biochemistry*.

[9] Wuertz K., Vo N., Kletsas D., Boos N. (2012). Inflammatory and Catabolic Signalling in Intervertebral Discs: The Roles of NF-κB and MAP Kinases. *European Cells and Materials*.

[10] Reilly S. M., Chiang S.-H., Decker S. J. (2013). An Inhibitor of the Protein Kinases TBK1 and IKK-ɛ Improves Obesity-Related Metabolic Dysfunctions in Mice. *Nature Medicine*.

[11] Zhang G.-Z., Liu M.-Q., Chen H.-W. (2021). NF-κB Signalling Pathways in Nucleus Pulposus Cell Function and Intervertebral Disc Degeneration. *Cell Proliferation*.

[12] Xie C., Kang J., Li Z. (2012). The Acai Flavonoid Velutin Is a Potent Anti-Inflammatory Agent: Blockade of LPS-Mediated TNF-α and IL-6 Production Through Inhibiting NF-κB Activation and MAPK Pathway. *Journal of Nutritional Biochemistry*.

[13] Wang K., Lu X., Li X. (2022). Dual Protective Role of Velutin Against Articular Cartilage Degeneration and Subchondral Bone Loss via the p38 Signaling Pathway in Murine Osteoarthritis. *Frontiers in Endocrinology*.

[14] Wang H., Ng T. B. (2001). Isolation and Characterization of Velutin, a Novel Low-Molecular-Weight Ribosome-Inactivating Protein From Winter Mushroom (*Flammulina velutipes*) Fruiting Bodies. *Life Sciences*.

[15] Jung S.-H., Kim J., Eum J. (2019). Velutin, an Aglycone Extracted From Korean Mistletoe, With Improved Inhibitory Activity Against Melanin Biosynthesis. *Molecules*.

[16] Yang X., Chen Y., Guo J. (2023). Polydopamine Nanoparticles Targeting Ferroptosis Mitigate Intervertebral Disc Degeneration Via Reactive Oxygen Species Depletion, Iron Ions Chelation, and GPX4 Ubiquitination Suppression. *Advanced Science*.

[17] Zhu H., Chen G., Wang Y. (2020). Dimethyl Fumarate Protects Nucleus Pulposus Cells From Inflammation and Oxidative Stress and Delays the Intervertebral Disc Degeneration. *Experimental and Therapeutic Medicine*.

[18] Cui S., Zhang L. (2021). MicroRNA-129-5p Shuttled by Mesenchymal Stem Cell-Derived Extracellular Vesicles Alleviates Intervertebral Disc Degeneration via Blockade of LRG1-Mediated p38 MAPK Activation. *Journal of Tissue Engineering*.

[19] Le Maitre C. L., Hoyland J. A., Freemont A. J. (2007). Interleukin-1 Receptor Antagonist Delivered Directly and by Gene Therapy Inhibits Matrix Degradation in the Intact Degenerate Human Intervertebral Disc: An In Situ Zymographic and Gene Therapy Study. *Arthritis Research & Therapy*.

[20] Wang Y., Che M., Xin J., Zheng Z., Li J., Zhang S. (2020). The Role of IL-1β and TNF-α in Intervertebral Disc Degeneration. *Biomedicine & Pharmacotherapy*.

[21] Madigan L., Vaccaro A. R., Spector L. R., Milam A. R. (2009). Management of Symptomatic Lumbar Degenerative Disk Disease. *Journal of the American Academy of Orthopaedic Surgeons*.

[22] Lyu F. J., Cui H., Pan H. (2021). Painful Intervertebral Disc Degeneration and Inflammation: From Laboratory Evidence to Clinical Interventions. *Bone Research*.

[23] Liang H., Luo R., Li G., Zhang W., Song Y., Yang C. (2022). The Proteolysis of ECM in Intervertebral Disc Degeneration. *International Journal of Molecular Sciences*.

[24] Xie G., Liang C., Yu H., Zhang Q. (2021). Association Between Polymorphisms of Collagen Genes and Susceptibility to Intervertebral Disc Degeneration: A Meta-Analysis. *Journal of Orthopaedic Surgery and Research*.

[25] Trefilova V. V., Shnayder N. A., Petrova M. M. (2021). The Role of Polymorphisms in Collagen-Encoding Genes in Intervertebral Disc Degeneration. *Biomolecules*.

[26] Zhang H.-J., Liao H.-Y., Bai D.-Y., Wang Z.-Q., Xie X.-W. (2021). MAPK/ERK Signaling Pathway: A Potential Target for the Treatment of Intervertebral Disc Degeneration. *Biomedicine & Pharmacotherapy*.

[27] Tam V., Chan W. C. W., Leung V. Y. L. (2018). Histological and Reference System for the Analysis of Mouse Intervertebral Disc. *Journal of Orthopaedic Research*.

